# The Role of Exposure History on HIV Acquisition: Insights from Repeated Low-dose Challenge Studies

**DOI:** 10.1371/journal.pcbi.1002767

**Published:** 2012-11-08

**Authors:** Roland R. Regoes

**Affiliations:** Integrative Biology, ETH Zurich, Zurich, Switzerland; Utrecht University, Netherlands

## Abstract

To assess the efficacy of HIV vaccine candidates or preventive treatment, many research groups have started to challenge monkeys repeatedly with low doses of the virus. Such challenge data provide a unique opportunity to assess the importance of exposure history for the acquisition of the infection. I developed stochastic models to analyze previously published challenge data. In the mathematical models, I allowed for variation of the animals' susceptibility to infection across challenge repeats, or across animals. In none of the studies I analyzed, I found evidence for an immunizing effect of non-infecting challenges, and in most studies, there is no evidence for variation in the susceptibilities to the challenges across animals. A notable exception was a challenge experiment by Letvin et al. Sci Translat Med (2011) conducted with the strain SIVsmE660. The challenge data of this experiment showed significant susceptibility variation from animal-to-animal, which is consistent with previously established genetic differences between the involved animals. For the studies which did not show significant immunizing effects and susceptibility differences, I conducted a power analysis and could thus exclude a very strong immunization effect for some of the studies. These findings validate the assumption that non-infecting challenges do not immunize an animal — an assumption that is central in the argument that repeated low-dose challenge experiments increase the statistical power of preclinical HIV vaccine trials. They are also relevant for our understanding of the role of exposure history for HIV acquisition and forecasting the epidemiological spread of HIV.

## Author Summary

Individuals are exposed to Human Immunodeficiency Virus (HIV) many times before they contract the virus. It is not known what an instance of exposure, which does not result in infection, does to the host. Frequent exposures to the virus are hypothesized to immunize an individual, and result in resistance to infection with HIV. This hypothesis may explain the resistance observed in some individuals despite frequent exposure to the virus. Since it is very difficult to monitor the HIV exposure and infection status of humans, this question is easier to address in animal models. I took data from previously published infection experiments of monkeys with Simian Immunodeficiency Virus (SIV) and analyzed them with newly developed mathematical models. I found that there is no evidence that challenging monkeys with the virus reduces their susceptibility to infection. These findings have important repercussions for the testing of HIV vaccines in monkeys, and also for our understanding of the role of exposure history for the acquisition of HIV.

## Introduction

Before being tested clinically, vaccines or preventive treatment strategies against human-immunodeficiency virus (HIV) are assessed in non-human primates. The paradigm of how vaccine candidates are assessed preclinically has been shifting in recent years. A decade ago, vaccine protection was not quantified directly by determining how much the vaccine candidate reduced the susceptibility of the animal hosts to infection. Instead, indirect measures were used, such as the level of virus-specific immune responses, or as the reduction of the viral set-point induced by vaccination.

Unfortunately, there are many uncertainties about how these immunological and virological measures correlate with protection. As a consequence, vaccines have recently been tested using repeated low-dose challenge experiments [1]–[13]. In such experiments animals are challenged repeatedly with doses of the virus, which do not give rise to infection with certainty. This protocol not just more realistically reflects the repeated exposure to HIV that human hosts face in the epidemic, but also allows the experimenter to directly measure the reduction of the hosts' susceptibility induced by the vaccine. Thus, repeated low dose challenge experiments are conceptually closer to clinical studies [14].

The challenge data generated in such trials are usually analyzed to infer the efficacy of a vaccine candidate, or post-exposure prophylaxis. In addition to information on treatment efficacy, however, these data contain information on other, very relevant aspects of the transmission of HIV. In the present study, I analyzed challenge data that had been published previously. I focused on the challenge data from control animals that did not receive any vaccine or treatment.

My first main question was if hosts are immunized by repeated challenges. In SIV challenge experiments, potential immunization is usually studied by measuring systemic or localized immune responses in an animal after a non-infecting challenge. This approach relied on the strong assumption that these immune measures are causatively linked to protection. To my knowledge, however, no such link has been systematically ascertained for SIV infection to date.

In this paper, I adopted an alternative approach to assess if immunization has occurred after challenge: I essentially compared the susceptibility of animals before and after challenge. Therefore, throughout this paper, *immunization* denotes a reduction of susceptibility that is brought about by non-infecting challenges. This definition of immunization does not require to know and measure the immune effector conferring protection, and directly quantifies what matters epidemiologically.

The second main question I posed was if there are differences in susceptibilities between animals. Again my approach focused on differences of animals in terms of their susceptibility to infection, and did not involve the quantification of target cells or their susceptibility in relevant anatomical sites.

As is common in mathematical epidemiology, I conceptualized infection as a stochastic event that occurs with a given probability. More precisely, I described infection as a Bernoulli process. While the infection probability can be thought of as a trait of an individual animal, its estimation requires data of more than one animal. Repeated low-dose challenge data allow us to estimate the infection probability. They also allow to test if animals are immunized by non-infecting challenges, as immunization leads to a smaller and smaller fraction of animals becoming infected in the course of the challenge experiment. The same is true if there are differences in susceptibility between animals. As I show below, one typically finds evidence for both, immunization and susceptibility differences, and further analysis is needed to disentangle the two effects. Formally, I used simple stochastic models and maximum likelihood estimation to estimate the infection probability, and to study how it varies across challenge repeats and animals.

I found that there is no evidence for immunization in any of the studies. There is also no evidence for variation in susceptibility, except for one recent experiment conducted with the strain SIVsmE660 [13]. In that study, genetic differences in susceptibility to SIVsmE660 had been previously established. Taken together, these results show that one of the central assumptions of the repeated low-dose challenge approach is not violated: there is no evidence that challenge history affects the probability of infection. The findings also have implications for our understanding of the role of repeated exposures in HIV acquisition.

## Results

**Figure 1:**
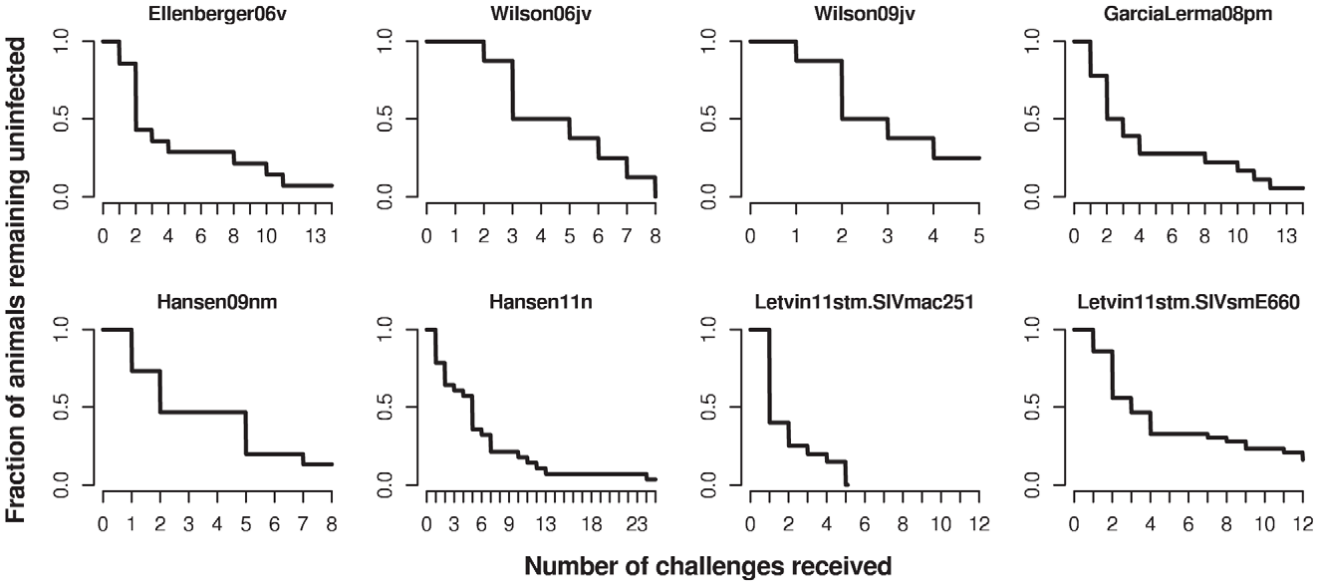
Challenge data of the control animals from the studies [5], [6], [8]–[10], [13], considered in this paper. The step functions for each study terminate at the maximum number of challenges applied in this study, or when all animals are infected.

I analyzed repeated low-dose challenge data from seven studies [5], [6], [8]–[10], [13], [15]. In brief, in these studies monkeys were challenged with SIV. The dose of the challenge was low, such that infection did not occur with certainty after a single challenge. The monkeys were challenged repeatedly in regular intervals of one or three weeks. The details of each individual dataset are described in the Materials and Methods section and shown in Table 1. Figure 1 summarizes the challenge data from the seven studies I analyzed.

**Table 1:** Summary of the experimental parameters of the studies analyzed.

	Monkey species	Number of monkeys (ctl/vac)	Challenge virus	Challenge route	Challenge frequency	Challenge dose	Maximum number of challenges	Treatment	Efficacy¶
**Ellenberger06v**	pigtailed macaques	14/16	SHIV-SF162P3	rectal	weekly	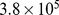 virus particles	up to 26	DNA/MVA vaccine	64%
**Wilson06jv**	rhesus macaques	8/8	SIVmac239	rectal	weekly	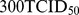 †	8	DNA/Ad5 vaccine	NS^0^
**Wilson09jv**	rhesus macaques	8/8	SIVsmE660	rectal	3 weeks	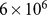 virus particles	5	DNA/Ad5 vaccine	NS^0^
**GarciaLerma08pm**	rhesus macaques	18/24	SHIV-SF162P3	rectal	weekly	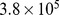 virus particles	14	FTC/TDF pre-exposure prophylaxis	74–87%
**Hansen09nm**	rhesus macaques	15/12	SIVmac239	rectal	weekly	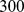 FFU‡	8	RhCMV/SIV vaccine	ND*
**Hansen11n**	rhesus macaques	28/33	SIVmac239	rectal	weekly	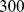 FFU‡	25	RhCMV/SIV & DNA/Ad5 vaccines	NS^0^
**Letvin11stm**	rhesus macaques	20/20	SIVmac251	rectal	weekly	1 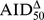	12	DNA/Ad5 vaccine	NS^0^
**Letvin11stm**	rhesus macaques	43/43	SIVsmE660	rectal	weekly	1 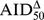	12	DNA/Ad5 vaccine	50%

† “Tissue culture infectious dose 50”;

‡ “focus forming units”;

Δ “animal infectious dose at which 50% become infected”;

¶ the efficacy of the intervention on susceptibility to challenge;

* not done;

0 not significantly different from 0%.

In the most recent study [13], genetic susceptibility differences were established for the challenge strain SIVsmE660. The gene implicated was TRIM5. Animals could be divided into “permissive” and “restricted” groups depending on the allele of TRIM5 they carried. (TRIM5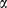 is a molecular factor that can block viral replication within cells.) The SIVsmE660 challenge data of that study will serve as a useful control for our methods to detect susceptibility differences.

### Investigating potential immunization

**Figure 2:**
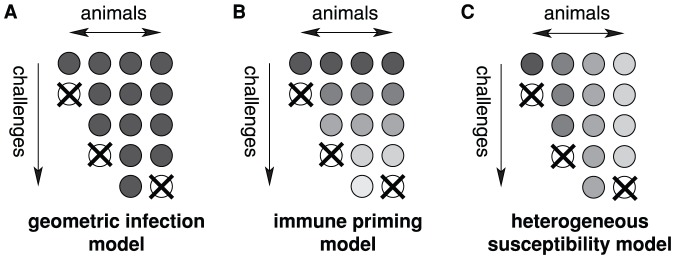
Diagrammatic representation of the infection models. **A** the geometric infection model, **B** the immune priming model, and **C** the heterogeneous susceptibility model. The circles represent animals, and the darkness corresponds to their susceptibility. Crossed circles signify that an animal has become infected. The geometric infection model assumes equal susceptibilities across animals and challenge repeats. In the immune priming model, the susceptibilities decrease with each challenge received, but animals that received the same number of challenges have the same susceptibility (illustrated by the same level of grey along the animal axis). In the heterogeneous susceptibility model, the susceptibility is assumed to vary across animals, but not with challenge repeats (illustrated by the same level of grey along the challenge axis).

**Figure 3:**
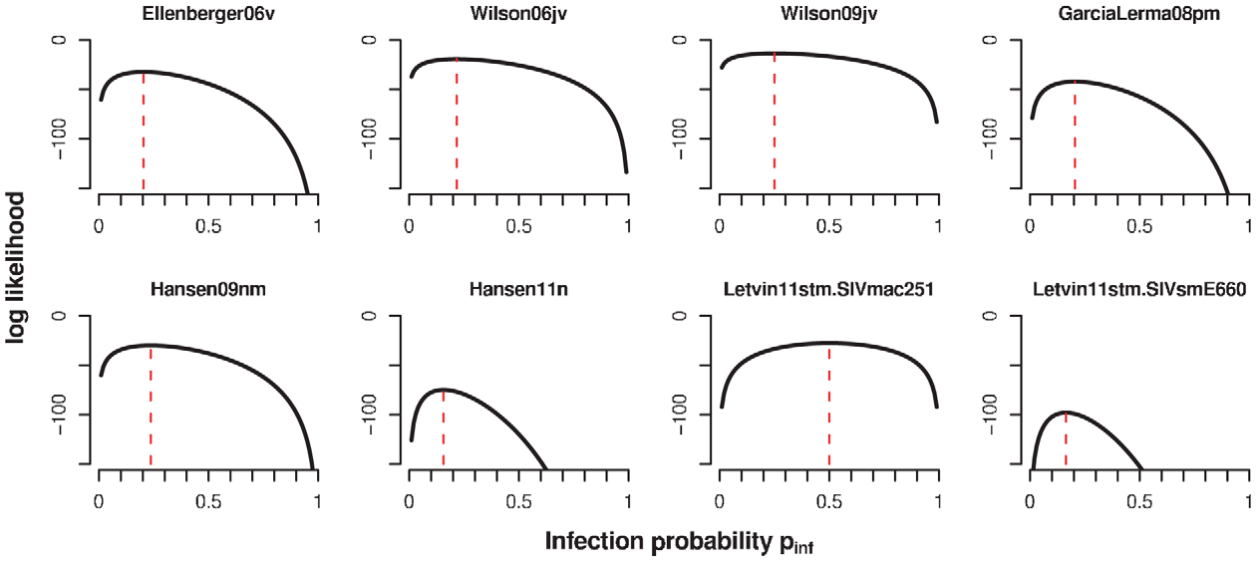
Likelihood of the geometric infection model for the different datasets. The red dashed lines indicates the maximum of the likelihood.

First, I assessed if there is evidence in the repeated low-dose challenge data for immunization in the sense that challenges, which do not give rise to infection, reduce suceptibility of the host. To this end, I first fit a stochastic model (the *geometric infection model*), which assumes that each animal has the same susceptibility to infection, 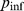, and that this susceptibility does not change from challenge to challenge (see see Figure 2**A** and Materials and Methods). This model served as a null model against which the more complex models are compared. The fit of the geometric infection model is summarized in Table 2, and a plot of the likelihoods as a function of 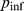 can be found in Figure 3. The estimated infection probability, 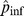, of each individual study ranges from 0.16 to 0.25 (see Table 2), except for the SIVmac251 challenge data in Letvin et al 2011, for which the estimate of 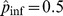.

**Table 2:** Fits of the geometric infection model.

	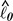	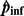	(95% CI)
Ellenberger06v	−32.3	0.20	(0.12, 0.31)
Wilson06jv	−19.3	0.22	(0.11, 0.37)
Wilson09jv	−13.5	0.25	(0.11, 0.44)
GarciaLerma08pm	−42.1	0.20	(0.13, 0.3)
Hansen09nm	−30.1	0.24	(0.14, 0.36)
Hansen11n	−74.9	0.16	(0.11, 0.21)
Letvin11stm.SIVmac251	−27.7	0.50	(0.35, 0.65)
Letvin11stm.SIVsmE660	−98.0	0.16	(0.12, 0.22)

In a second step, I fit the immunization model to the data. This model assumed that the susceptibility to the challenge decreased with challenge repeats (see Figure 2**B**). I considered various ways in which such a decrease could occur. The susceptibility could drop after the first challenge from a value, 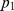, to a lower value, 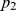. Alternatively, it could drop to this lower level after the 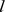
th challenge, rather than the first. Lastly, the susceptibility may start at a value 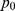, and change incrementally by a fixed amount 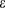 (see Materials and Methods). While these models certainly do not comprise every conceivable immunizing effect they serve as a good compromise between what is conceivable immunologically and the mathematical simplicity of their description. Without this simplicity one would lose statistical power.

**Figure 4:**
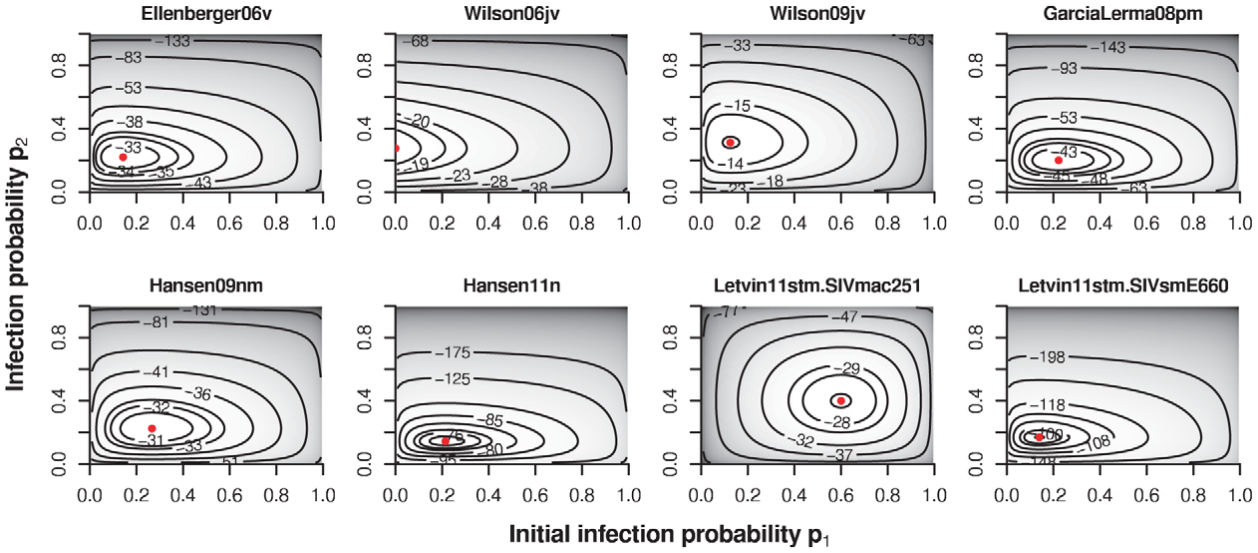
Contour plots of likelihood of the immune priming model (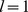) for the different datasets. The red dots indicate the maximum of the likelihood.

Irrespective of the way I implemented immunization, the immune priming model fails to outperform the geometric infection model statistically, except for the SIVsmE660 challenge data of Letvin et al 2011 (in which susceptibility difference between the monkeys have been established), and Wilson et al 2006. Table 3 shows the maximum log-likelihoods, 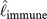, for the immune priming model in its various formulations, along with maximum likelihood estimator for the parameters 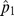 and 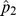, or 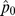 and 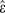 for the incremental immune priming model variant. The likelihoods of this model are smooth and have clearly defined global maxima (see Figure 4). Table 3 also shows the 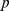-value of a likelihood ratio test against the geometric infection model. In most cases, none of the immune priming model variants explain the data better than the geometric infection model.

**Table 3:** Statistical comparison of the immune priming models to the geometric infection model.

	immunization	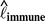	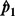 or 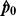 (95% CI)	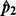 or 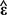 (95% CI)	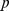 -value
Ellenberger06v	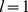	−32.1	0.14 (0.03, 0.38)	0.22 (0.12, 0.35)	0.51
	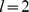	−30.8	0.31 (0.15, 0.5)	0.13 (0.05, 0.26)	0.09
	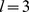	−31.1	0.28 (0.15, 0.45)	0.12 (0.04, 0.27)	0.12
	incremental	−31.9	0.25 (0.12, 0.41)	−0.01 (−0.03, 0.02)	0.36
Wilson06jv	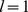	−17.1	0.00 (0, 0.21)	0.28 (0.14, 0.45)	0.03 *
	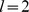	−17.1	0.06 (0, 0.25)	0.33 (0.16, 0.55)	0.04 *
	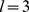	−19.0	0.17 (0.06, 0.36)	0.29 (0.1, 0.54)	0.43
	incremental	−16.5	0.00 (0, 0.21)	0.09 (0.02, 0.14)	0.02 *
Wilson09jv	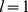	−13.0	0.13 (0, 0.45)	0.31 (0.13, 0.56)	0.30
	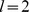	−13.5	0.27 (0.09, 0.52)	0.22 (0.04, 0.54)	0.81
	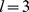	−13.5	0.26 (0.1, 0.48)	0.20 (0.01, 0.63)	0.77
	incremental	−13.5	0.25 (0.04, 0.54)	0.00 (−0.14, 0.15)	1.00
GarciaLerma08pm	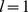	−42.1	0.22 (0.07, 0.44)	0.20 (0.12, 0.31)	0.84
	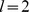	−41.2	0.28 (0.15, 0.45)	0.16 (0.07, 0.27)	0.18
	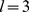	−41.1	0.27 (0.15, 0.42)	0.14 (0.06, 0.27)	0.15
	incremental	−41.8	0.24 (0.13, 0.38)	−0.01 (−0.03, 0.01)	0.43
Hansen09nm	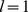	−30.0	0.27 (0.09, 0.52)	0.22 (0.12, 0.37)	0.75
	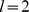	−29.4	0.31 (0.15, 0.5)	0.17 (0.07, 0.33)	0.24
	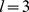	−30.1	0.24 (0.12, 0.4)	0.23 (0.09, 0.43)	0.90
	incremental	−30.0	0.26 (0.12, 0.45)	−0.01 (−0.06, 0.04)	0.69
Hansen11n	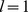	−74.5	0.21 (0.09, 0.39)	0.14 (0.09, 0.21)	0.37
	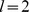	−74.4	0.20 (0.11, 0.32)	0.14 (0.08, 0.21)	0.32
	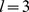	−74.9	0.16 (0.09, 0.26)	0.15 (0.09, 0.23)	0.87
	incremental	−74.3	0.18 (0.11, 0.26)	−0.00 (−0.01, 0)	0.27
Letvin11stm.SIVmac251	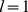	−26.9	0.60 (0.38, 0.79)	0.40 (0.21, 0.62)	0.20
	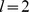	−27.5	0.54 (0.35, 0.71)	0.42 (0.17, 0.69)	0.49
	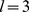	−27.6	0.48 (0.32, 0.65)	0.57 (0.23, 0.87)	0.68
	incremental	−27.7	0.52 (0.32, 0.71)	−0.01 (−0.12, 0.11)	0.81
Letvin11stm.SIVsmE660	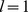	−97.9	0.14 (0.06, 0.26)	0.17 (0.12, 0.23)	0.63
	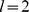	−95.6	0.24 (0.15, 0.34)	0.12 (0.07, 0.18)	0.03 *
	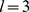	−95.6	0.22 (0.15, 0.31)	0.11 (0.06, 0.18)	0.03 *
	incremental	−96.4	0.21 (0.14, 0.29)	−0.01 (−0.02, 0)	0.07
Letvin11stm.SIVsmE660	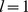	−54.5	0.15 (0.05, 0.31)	0.32 (0.22, 0.43)	0.08
(permissive TRIM5 alleles)	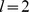	−56.1	0.28 (0.17, 0.41)	0.26 (0.15, 0.40)	0.83
	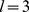	−56.0	0.29 (0.18. 0.40)	0.24 (0.12,0.40)	0.65
	incremental	−55.9	0.29 (0.18,0.42)	−0.01 (−0.03,0.02)	0.55
Letvin11stm.SIVsmE660	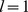	−34.5	0.13 (0.02, 0.34)	0.07 (0.03, 0.13)	0.51
(restrictive TRIM5 alleles)	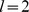	−33.1	0.17 (0.06, 0.32)	0.05 (0.02, 0.11)	0.06
	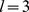	−34.1	0.12 (0.05, 0.24)	0.06 (0.02, 0.13)	0.25
	incremental	−34.7		−0.00 (−0.01, 0.01)	0.66

CI abbreviates confidence interval. The last column gives the 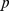-values for a likelihood ratio test, and significant tests are marked by ^*^.

A notable exception are the data by Wilson et al, J Virol 2006. [6]. It is important to note, however, that the effect in these data is the opposite of immunization: the susceptibility increases with challenges. This result is due to the fact that none of the animals in the experiments by Wilson et al, J Virol 2006 [6] became infected at the first or second challenge. Therefore, the susceptibility at first and second challenge is estimated as 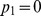 for the models with 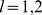, and the incremental variant of the immune priming model 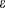 is estimated to be positive.

I also found a significant improvement of the fit of the immune priming models over the geometric infection model for the SIVsmE660 challenge data of Letvin et al 2011. In particular, the immune priming model with an approximately two-fold drop in susceptibility after the second or third challenge (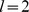 or 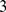) improved the fit significantly over the geometric infection model (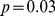). However, this can be attributed to the susceptibility differences between the monkeys in that dataset. Repeating the analysis on the subgroups carrying permissive and restrictive TRIM5 alleles separately, yielded no evidence for immunization (see Table 3). In summary, there is no evidence for immunization by viral challenges in these datasets.

### Investigating potential differences in animal susceptibility

To assess if there is any evidence for differences in susceptibility between animals, I followed the same statistical approach as in the previous subsection: I compared the fit of the geometric infection model to that of a model, in which the susceptibilities are allowed to vary from animal to animal (the *heterogeneous susceptibility model*). The heterogeneous susceptibility model is mathematically defined in the Materials and Methods section and diagrammatically shown in Figure 2**C**. This model has two parameters, one for the mean infection probability, 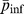, and another measuring the variance in susceptibilities across animals, 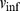.

**Figure 5:**
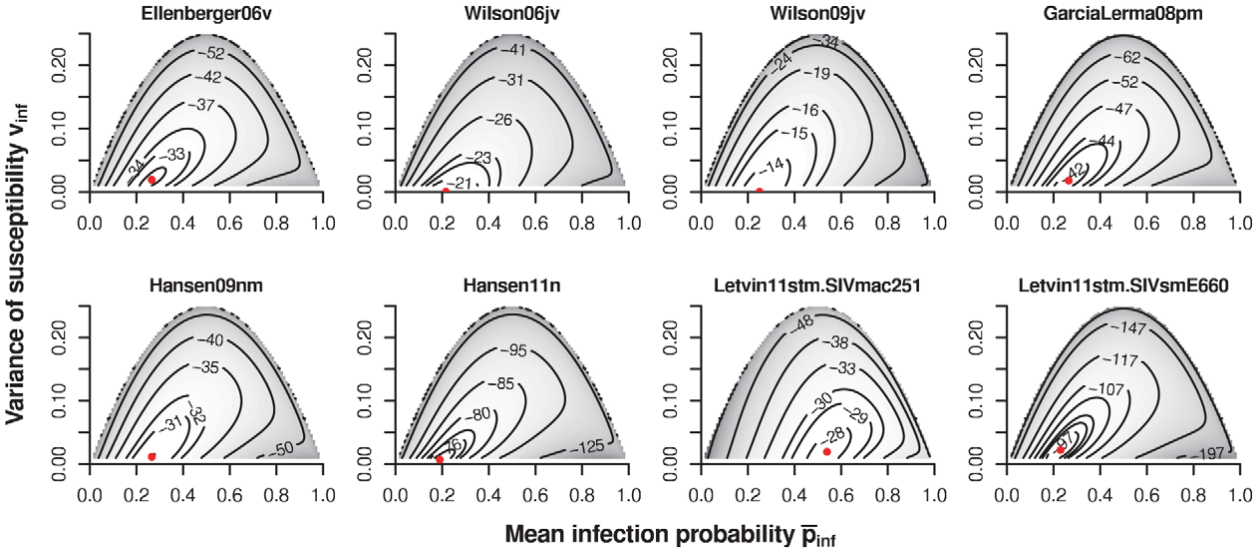
Contour plots of likelihood of the heterogeneous susceptibility model for the different datasets. The red dots indicate the maximum of the likelihood.

The likelihoods as a function of the two parameters of this model are shown in Figure 5 for each of the datasets analyzed. Table 4 shows the maximum log-likelihood, 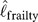, the maximum likelihood estimators, 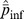 and 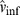, and the 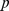-values for a likelihood ratio test against the geometric infection model fit (see Table 2) for the heterogeneous susceptibility model.

**Table 4:** Statistical comparison of the heterogeneous susceptibility model to the geometric infection model.

	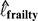	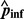	(95% CI)	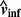	(95% CI)	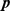 -value
Ellenberger06v	−31.8	0.27	(0.13, 0.48)	0.019	(0, 0.094)	0.32
Wilson06jv	−19.3	0.22	(0.11, 0.37)	0.000	(0, 0.031)	1.00
Wilson09jv	−13.5	0.25	(0.11, 0.53)	0.000	(0, 0.123)	1.00
GarciaLerma08pm	−41.6	0.26	(0.14, 0.46)	0.018	(0, 0.088)	0.32
Hansen09nm	−30.0	0.27	(0.14, 0.5)	0.011	(0, 0.106)	0.67
Hansen11n	−74.3	0.19	(0.11, 0.31)	0.007	(0, 0.043)	0.27
Letvin11stm.SIVmac251	−27.6	0.54	(0.35, 0.76)	0.019	(0, 0.11)	0.70
Letvin11stm.SIVsmE660	−96.0	0.23	(0.15, 0.34)	0.022	(3e−04, 0.065)	0.04 *

CI abbreviates confidence interval. The last column gives the 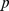-values for a likelihood ratio test, and significant tests are marked by ^*^.

For the dataset for which susceptibility difference have been established (Letvin11stm.SIVsmE660), I found significant levels of inter-animal susceptibility differences (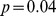). This shows that the statistical approach I adopted works. It further shows that susceptibility differences can be established without having to know their molecular or genetic basis. (My analysis did not use information on the TRIM5 alleles that the animals carried.) For none of the other datasets does the heterogeneous susceptibility model fit better than the geometric infection model, and hence there is no evidence for variability of susceptibilities between animals.

### Power of the experiments

The absence of evidence must not be confused with the evidence of absence. The non-significant results in the previous two subsections could simply be due to low sample sizes. To address this possibility, I conducted a power analysis. I simulated experiments using the same number of animals and challenges as in the experimental data, assuming immunization effects or inter-animal susceptibility differences of various sizes. I then analyzed these simulated data to test for immunization or heterogeneous susceptibility (see Materials and Methods for more detail).

**Figure 6:**
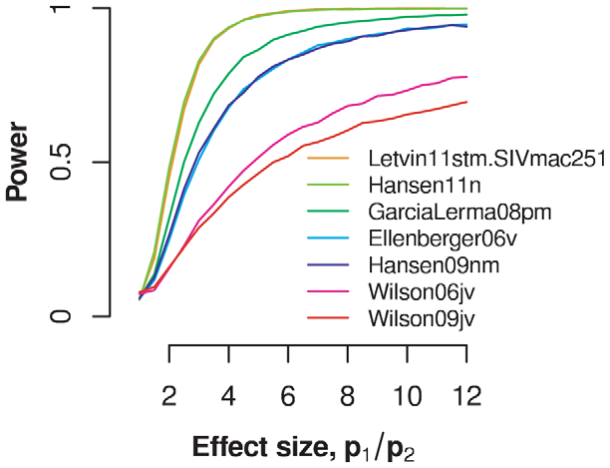
Power of the experiments to establish an immunization effect. Power estimates are shown as a function of the effect size. The *in silico* challenge data are generated according to an immune priming model in which the infection probability drops from an initial value of 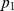 to a lower value 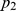 after the first challenge (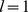). Effect size is measured as the ratio between 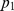 and 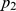. The different curves are generated with the same number of animals and maximum challenge repeats as the datasets indicated in the legend.

For the immunization model, I defined the effect size as the relative reduction of susceptibility after the first challenge, 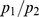. Figure 6 shows the result of this analysis. The least powerful experiment is that by Wilson et al, J Virol 2006 [6] because it involves only eight control animals and at most eight challenges. The most powerful experiments are those conducted with SIVmac251 by [13] and those by [15]. The first experiment involved 20 animals challenged at most 12 times, the second 28 animals challenged at most 25 times. For these experiments, the probability not to uncover a significant immunization effect is less than 5% for an effect size of 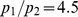, i.e. if the susceptibility was reduced by a factor of approximately 4.5 after the first unsuccessful challenge. A four-fold reduction still constitutes a large immunization effect.

How likely is it that I missed a significant effect in all of the studies simultaneously? A power analysis, in which I simulated each study repeatedly assuming study-specific model parameters (see Materials and Methods), yielded that the probability to miss an immunization effect in all these studies of size 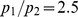 is less than 5%. This analysis is valid only if an immunization effect is present and equal across all the early studies.

For the heterogeneous susceptibility model, the effect size was defined as the variance of the susceptibility distribution. Depending on the variance, the shape of the susceptibility distribution can be hump-shaped, monotonously falling (or rising), or U-shaped. The maximum variance depends on the mean of the distribution. For example, for a mean infection probability of 0.2 — the most common estimate obtained by fitting the geometric infection model to the various datasets — this maximum is 0.16. For Letvin11stm.SIVmac251, however, the mean infection probability is 0.5, and the maximum possible variance is 0.25.

**Figure 7:**
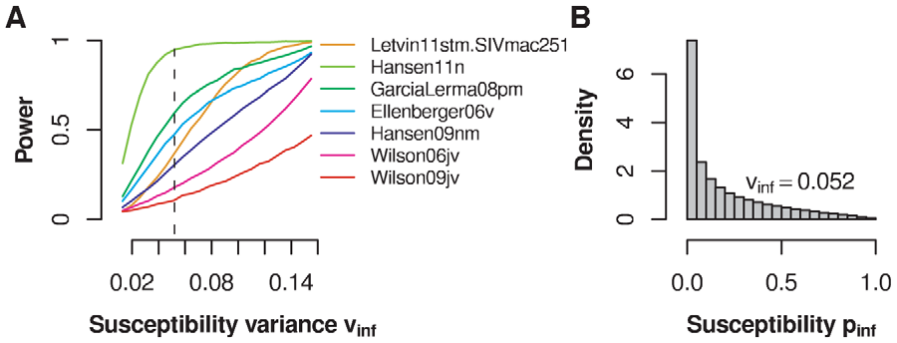
Power of the experiments to establish susceptibility differences between animals. **A** Power estimates are shown as a function of the effect size measured by the variance parameter 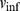. The different curves are generated with the same number of animals and maximum challenge repeats as the datasets indicated in the legend. The dashed line shows the level of susceptibility variance (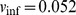), for which an experiment with the same number of animals and maximum challenge repeats as the one in Hansen et al (2011) has a power larger than 0.95. **B** Histogram of a susceptibility distribution with 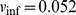. A susceptibility distribution more heterogeneous than the one shown can be ruled out with 95% probability.

Figure 7**A** shows the result of a power analysis for various levels of heterogeneity in animal susceptibility. The power to establish susceptbility differences between animals differs for each individual study. The dataset by Hansen et al 2011 has the highest power, and can be used to exclude a level of heterogeneity 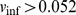. The shape of this critical susceptibility distribution with 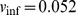 is shown in Figure 7**B**. The critical 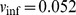 describes a monotonously falling susceptibility distribution with large differences in heterogeneity between animals. For this dataset, one can exclude only the largest conceivable heterogeneity: a U-shaped susceptibility distribution describing a scenario according to which approximately one fifth of the animals are almost completely susceptible and the remaining animals are alomost completely resistant to infection.

Again, one can ask how probable it is that we missed a significant effect in all of the studies simultaneously. The probability not to detect susceptbility differences in any of the early studies is lower than 5% for susceptibility differences larger than 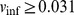. This analysis assumed equal levels of heterogeneity across the different studies.

## Discussion

Using challenge data that were generated in the context of preclinical HIV vaccine studies in non-human primates, I investigated if low-dose challenges immunize the animal hosts. Potential immunization has been raised as an argument against the repeated low-dose challenge approach, which could impair its statistical power advantage. I also studied if there is evidence for susceptibility differences between animals. Formally, the analysis involved fitting simple stochastic models to the challenge data. To establish immunization or heterogeneity in susceptibility, the fits of models that accounted for such effects were compared statistically to fits of a model that ignored them.

For none of the datasets, I found evidence for immunization. There is also no evidence for differences in susceptibilities, except in the SIVsmE660 challenge data presented in [13], in which susceptibility differences have been previously identified. For SIVsmE660 it had been established that an animal's susceptibility depends on the TRIM5 alleles it carries. TRIM5 encodes for the restriction factor Trim5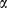, which is thought to interact with the capsid of the virus after it has infected a cell. Letvin et al show that monkeys that carry exclusively TRIM5 alleles classified as “restrictive” have a reduced susceptibility to infection as compared to monkeys that carry “permissive” alleles. Comparing the fit of the geometric infection model to that of the heterogeneous susceptibility models, I found significant levels of heterogeneity in these challenge data.

It is important to emphasize that the evidence for susceptibility differences in this dataset is not based on information of the TRIM5 alleles the animals carry. The inference only relies on the distribution of the number of challenges across animals. Hence, the method I am presenting allows the identification of heterogeneity in susceptibility from the challenge data alone and does not rely on measuring traits that modulate susceptibility. Such a factor ignorant method is important tool as considerable uncertainties about the determinants of susceptibility remain.

The SIVsmE660 challenge data presented in [13] are also consistent with an immunization effect, although not for every type of immune priming I considered. This finding, however, is very likely an artefact of the statistical approach known as one of “hetergeneity's ruses” [16]. Both effects — immunization and susceptibility differences — manifest themselves by an overdispersion of the challenge data as compared to a geometric distribution. Immunization decreases the susceptibility to late challenges due to the immune responses elicited by early challenges. A similar pattern arises if susceptibilities among the animals differ, but for a different reason. In this case, early challenges will more likely lead to infection of animals with higher susceptibility. This results in more animals with lower susceptibility late in the challenge schedule as these animals are more likely to remain uninfected. An immunization effect could therefore be incorrectly inferred from challenge data that arise from hosts that vary in their susceptibility.

In general it is difficult to disentangle the two effects. The difference between immunization and host heterogeneity is too subtle to be detected with the sample sizes of the challenge data I analyzed, and depends sensitively on the quantitative details of immunization effects and heterogeneity. However, as the SIVsmE660-challenged animals of the study by Letvin et al had been classified with respect to their susceptibility, I could test for immunization within these subgroups. As I did not find any evidence for immunization in each susceptibility class, I concluded that the immunization effect in the pooled data is misidentified.

The lack of evidence for immunization does, of course, not prove that there is no such effect. It may simply result from the low sample sizes in these studies. To go beyond this plain caveat, I conducted a power analysis that quantifies the probability that an effect was missed.

The study by Wilson et al (2009) may provide a likely case of too low power. This study used the same challenge strain (SIVsmE660) as the data by Letvin et al 2011, in which susceptibility differences between animals have been established. The animals involved in study by Wilson et al (2009) were, to my knowledge, not monitored for their TRIM5 alleles, but it is conceivable that some animals differed in their susceptibility for this reason. The power of this study, however, was the lowest among all the studies. To detect the same level of heterogeneity as I found in Letvin11stm.SIVsmE660 (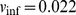) the power of the study by Wilson is below 10%.

While sample sizes were clearly an issue in the study by Wilson et al (2009), especially the later studies [13], [15] involved substantially larger numbers of animals. According to the power analysis, these studies allow the detection of immunization effect, albeit only large ones. Additionally, it is important to note that my power estimates are optimistic as the simulated data for the power analysis were generated with the same model as was used for the statistical analysis. The model describing the true immunization effect is likely to be different from the immune priming model, and this model misspecification will generally lead to lower power.

The lack of evidence for immunization by non-infecting challenges in the majority of the studies constitutes a crucial validation of the repeated low-dose challenge approach. Only if challenges do not immunize, one can safely assume that infection probabilities are independent. According to my analysis, there is no evidence against the assumption of independence. While we cannot exclude immunization effects of small size, the analysis presented in this paper provides evidence against at least very strong immunization effects. This suggests that the repeated low-dose challenge approach increases statistical power as we and others have previously predicted [14], [17].

Independently from the findings I present in this paper, the statistical power is further corroborated by the increasing number of studies that have used this approach successfully. For example, Ellenberger et al, Virol 2006 [5] established an efficacy of 64% of a DNA/MVA vaccine with 30 animals, and Garcia-Lerma et al, PLoS Med 2008 [8] could establish a treatment efficacy of 74–87% of pre-exposure prophylaxis with antivirals with 42 animals (see Table 1). To harvest the full power of the repeated low-dose approach it is further necessary to keep the time between challenges large enough to allow the identification of the challenge which gave rise to infection. Identification of the infecting challenge may have been the problem in the study by Wilson et al, J Virol 2006 [6] in which no animal was infected before the third challenge.

The power analysis also suggests that the susceptibility distribution among the experimental animals is, with high probability, not U-shaped. This is also very relevant to how vaccine efficacies are estimated statistically, and how many animals have to be involved in a preclinical study. If the susceptibility distribution were U-shaped, the animal population would essentially fall into two classes: almost completely susceptible and almost completely resistant. Any effect of a vaccine would be confined to the susceptible subpopulation, thus effectively decreasing the sample size.

But even in the case in which the susceptibility distribution is not U-shaped, yet susceptibilities still vary from animal to animal, some of the standard assumptions made when estimating vaccine efficacies from repeated low-dose challenge experiments are violated. While some studies consider animal-to-animal variation in the effect of the vaccine [18], the susceptibility of unvaccinated animals is most commonly assumed not to differ across animals. In future repeated low-dose challenge trials, I suggest to first check if there is evidence for susceptibility differences using the statistical approach presented in this paper. If this turned out to be the case frailty approaches should be adopted along the lines of [19] to estimate vaccine efficacies.

Beyond the context of assessing HIV vaccines or prophylaxis, repeated low-dose challenge data provide insights into the natural transmission of HIV. It is extra-ordinarily challenging to assess how the rate of HIV acquisition depends on the exposure history. The reason for this difficulty is that, on logistic grounds, individuals cannot be monitored frequently enough to generate exposure and acquisition data with the level of detail required to establish the role of exposure history.

For example, the Rakai cohort [20], [21], which provides some of the best data to estimate HIV transmission rates, involves approximately 200 HIV discordant couples. Individuals in this cohort are monitored every 10 months, and report on average 10 sex acts per month. Thus, individuals are tested for infection every 100 exposures on average, which does not allow to estimate reductions in susceptibility that are likely to be most pronounced during the first exposures to the virus. For these reasons there is no quantitative understanding of the dependence of the rate of HIV acquisition on exposure history to date.

Consequently, most mathematical models that forecast the epidemiological spread of HIV neglect exposure history and assume that hosts retain no memory of previous exposures. The findings in this paper provide limited support for this assumption. The support is only limited because of issues relating to statistical power mentioned above, but also because the doses used in repeated low-dose challenge experiments are still much higher than those transmitted naturally. To definitively rule out any impact of exposure history it will be necessary to conduct experiments in which the challenge dose is further reduced and the frequency is systematically varied from more often than daily to less often than weekly. Some immunization effect may not be detectable if hosts are exposed weekly, as was done in most of the studies I analyzed in the present paper.

There is a group of HIV exposed individuals — sex workers from Kenya and Uganda — who remain uninfected despite frequent exposure to the virus. These highly exposed seronegative (HESN) individuals are hypothesized to be immunized by frequent exposures to the virus [22]. It has been realized fifteen years ago that non-human primate models may provide a way to test for potential resistance due to exposure to the virus. However, early studies of this issue remained equivocal [23], [24]. These early studies on the role of exposure history also employed very high doses to challenge the monkeys. At these high challenge doses the experiments may not have been sensitive enough to detect resistance mechanisms that protect against naturally-occurring low-dose exposure.

The repeated low-dose challenge of monkeys much better reflects the frequent exposure of the HESN individuals, although the doses used in repeated low-dose challenge experiments are still high when compared to the doses to which humans are exposed. (They are termed “low” to distinguish them from the very high doses normally used in non-human primate challenge studies.) Therefore, if frequent exposure by itself were sufficient to lead to resistance, at least partial immunization should be observed in the challenge experiments. The fact that I failed to find any immunization effect suggests that there is more to the resistance of HESN individuals than high and frequent exposure. It is conceivable that the exposure frequency or dose is required to start at a low level and increase over time. The exposure route may also be relevant: in the studies I analyzed the challenge was performed rectally, while HESN individuals are exposed vaginally. A last possibility is that the frequency of challenges in the most of the experiments of one week is too low to kick off the immunizing mechanism, which render HESN individuals resistant. In any case, the hypotheses about resistance in HESN individuals will have to be refined by specifying the routes of infection as well as the ranges of exposure dose and frequency that can lead to resistance.

An important conclusion from the analysis presented in this paper is that the challenge data in every study — with the exception of the one by Letvin et al using SIVsmE660 as a challenge strain — are consistent with the geometric infection model. This means there is no evidence that animals differ in their susceptibilities. As a consequence, there is no justification to divide the animals in these studies into those that become infected early versus those become infected late in the challenge schedule. Neither is there any justification to compare these two groups immunologically, virologically or genetically. Approaches, such as the statistical comparison between the fit of geometric infection model with a fit of the heterogeneous susceptibility model, are required to establish susceptibility differences and to provide a solid statistical foundation for comparisons between animals with low and high susceptibility.

## Materials and Methods

### Experimental data

I selected repeated low-dose challenge data from seven previously published studies. These studies are: Ellenberger et al, Virol 2006; [5], Wilson et al, J Virol 2006; [6], Wilson et al, J Virol 2009; [10], Garcia-Lerma et al, PLoS Med 2008; [8], Hansen et al, Nat Med 2009; [9], and Hansen et al, Nature 2011; [15], and Letvin et al, Sci Transl Med 2011 [13]. The criteria for this selection were a sufficiently high number of monkeys involved, more than five challenge repeats applied, and the regularity of the challenge schedule.

Table 1 summarizes the most important aspects of the data. The monkey hosts were rhesus or pigtailed macaques, and the challenge virus was either the standard challenge strains SIVmac239 or SIVmac251 the sooty mangabey virus SIVsmE660, or SHIV-SF162P3, a chimera between SIV and HIV featuring a CCR5 tropic envelope protein [25].

The number of animals involved in the studies ranged from 16 to 86. The maximum number of challenges ranges from 8 to 26. Challenges were given rectally with a frequency of one week. Rectal challenges are the preferred route in such experiments as they can be performed on male animals and are relevant for human transmission. The involvement of female animals in preclinical studies is rare as they are required to maintain the colonies.

I analyzed only challenge data of the control animals involved in the studies listed in Table 1. In these animals the susceptibility was not manipulated, and is thus most relevant to study the effects of exposure history.

In some studies the challenge dose was increased after a certain number of challenges. I ignored the data generated with increased doses. The reason for this is that the challenge with increased doses pertained to only few animals, and would therefore be only marginally informative. Moreover, incorporating these data would have forced me to introduce an additional susceptibility parameter into my models, which — due to the low sample size — could not be reliably estimated.

The dataset by Letvin et al (2011) involving challenges with the viral strain SIVsmE660 will serve as a control for our approach to establishing susceptibility differences. For this strain, genetic correlates of susceptibility have been identified (see Results and Discussion).

The challenge data consist of two pieces of information for each animal. The first is the number of challenges, 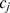, the 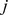
th animal received, and the second is the infection status 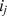 of this animal after the 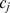 challenges. Hereby, 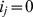 means that the animal remained uninfected, and 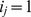 means that the animal was infected after receiving 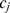 challenges. Note that in these experiments an animal that is found infected is not given any challenges anymore.

I constructed stochastic models and used them in combination with the challenge data to infer parameters characterizing the probability of animals becoming infected upon challenge with the virus. In the next subsection, I describe these models.

### Mathematical models

#### Geometric infection model

The simplest model I considered assumes that a challenge results in infection with a probability constant across animals and challenge repeats (see Figure 2**A**). This model predicts that the number of challenges that were required to infect the animals are geometrically distributed. Therefore I call it the *geometric infection model*.

Assume we have conducted a repeated challenge experiment with 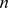 animals. I denote the probability that a single challenge gives rise to infection in a single animal by 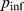. Further, let 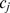 be the challenges animal 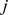 received, and 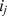 the infection status (0 = uninfected or 1 = infected) of animal 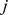 after 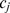 challenges. The likelihood of this experimental outcome is:
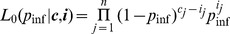
(1)


The probability of infection 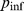 was estimated by maximizing the log-likelihood:
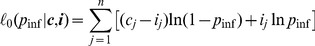
(2)Confidence intervals of the estimate of 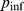 were obtained by calculating the “likelihood ratio confidence region” [26]:
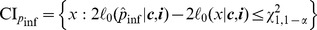
(3)Hereby, 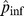 denotes the value of the infection probability that maximizes the likelihood, and 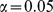 is the significance level.

#### Immune priming model

After a challenge that did not give rise to infection the host's susceptibility may be reduced due to its immunization by the unsuccessful challenge (see Figure 2**B**). To accommodate this possibility in the stochastic model of infection, I introduced the infection probabilities, 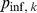, denoting the infection probability of the 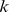
th challenge. These probabilities are assumed to be the same for each animal.

For challenge data 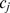 and 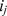 of the 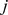
th animal 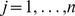, I obtained the following likelihood:
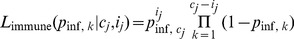
(4)The log-likelihood for the data of all animals is then:
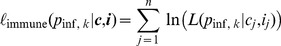
(5)


While this formulation allows any pattern of change of the infection probability 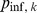 with challenge repeats, I considered two particular formulations. First, I assumed that the infection probability drops (or even increases) from 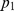 to 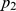 after the 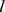
th challenge:
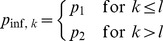
(6)In the second formulation that I considered, the infection probability decreases (or increases) incrementally from challenge to challenge:
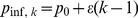
(7)


The 95% confidence interval for the estimate of each model parameter was calculated as the likelihood ratio confidence region of the parameter using the profile likelihoods for this parameter [26]. For the parameter 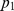, the confidence interval was calculated as:
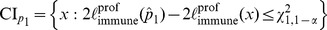
(8)In this expressions, 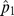 denotes the maximum likelihood estimate of 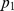, and the significance level 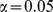. Further, the profile likelihood 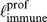 for 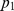 is given by:
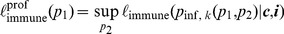
(9)Confidence intervals for the other parameters are defined analogously.

#### Heterogeneous susceptibility model

In this model, I assumed that the susceptibilities of each animal in the trial differ (see Figure 2**C**). As in the geometric infection model, I assumed that infection probabilities do not vary across challenge repeats. Instead of estimating a susceptibility for each animal individually, which would force us to estimate too many parameters, I adopted an approach known as *frailty modelling* from survival analysis, in which the probabilities of infection are drawn from a Beta distribution with parameters 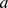 and 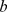. The mean infection probability across animals is given as 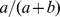, while the variance is 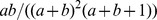.

To obtain the likelihood for one animal, which has been challenged 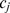 times and has infection status 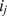, we have to calculate a weighted average over the Beta-distributed infection probability 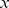:
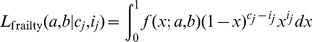
(10)

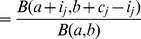
(11)Hereby, 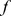 is the probability density distribution of the 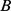-distribution and 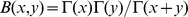 is the Beta function. The log-likelihood for the data of all animals is then:
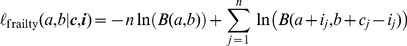
(12)


It is useful to re-parametrize this function since both parameters, 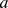 and 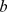, affect the mean and the variance of the susceptibility distribution. As new parameters I introduced the mean susceptibility, 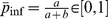, and the variance of the susceptibility, 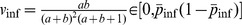. The re-parametrized log-likelihood is:
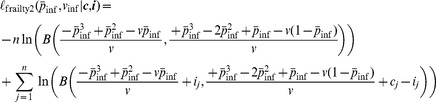
(13)


As for the immune priming model, the 95% confidence interval for the estimate of each model parameter was calculated as the likelihood ratio confidence region of the parameter using the profile likelihoods for this parameter [26].

### Model comparisons

To test for immunization by repeated challenges or for differences in the susceptibilities of animals to infection, I first fit the geometric infection model, and then the immune priming and heterogeneous susceptibility models. The model fits were then compared by a likelihood ratio test. I applied a significance level of 0.05.

### Power analysis

To determine the statistical power of the model fitting and comparison, I simulated data that conform to the immune priming or heterogeneous susceptibility models. In these simulations, I chose numbers of animals and maximum numbers of challenge repeats consistent with each experimental study.

In the case of the immune priming model, I set an animal's susceptibility at the first challenge is 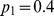 for all studies except for Letvin11stm.SIVmac251 for which I set 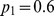. This probability was reduced after the first challenge by a factor ranging from 1 ( = no effect) to 12. In the simulation according to the heterogeneous susceptibility model, I set the mean probability of infection of 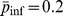 for all studies except for Letvin11stm.SIVmac251 for which I defined 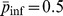. I further set the variance parameter 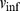 to values ranging from 0 ( = no effect) to 0.155. The value 0.16 is the maximum variance possible for a Beta distribution with mean 0.2.

The simulated data were then analyzed and significance was assessed. Power was determined as the fraction of simulated experiments, in which a significant immunization effect or heterogeneous susceptibilities could be established. In accordance with the comparison of the model fits to the experimental data, I applied a significance level of 0.05. If a simulated dataset could not be fitted (due to convergence problems of the fitting routine) it was excluded from the analysis.

### Implementation

The likelihoods, the model fitting and comparison, and the power analysis were implemented in the R language of statistical computing [27]. The datasets, implementations of the likelihood functions, and routines for the power analysis are provided as an R package in Protocol S1 and documented in Text S1.

## Supporting Information

Protocol S1 **R-package containing the datasets, likelihood and power analysis functions.** Once downloaded, the file can be installed in R [27] by executing install.packages(“<path to downloaded .tar.gz file>”, repos = NULL, type = “source”) from the R-prompt. Then load the package by executing library(“regoes12pcb”). Get started by calling the main manual page: ?regoes12pcb.
(GZ) Click here for additional data file.

Text S1 **Documentation of the R-package in pdf format.**
(PDF)
