# Supplementary material for: The Role of Exposure History on HIV Acquisition: Insights from Repeated Low-dose Challenge Studies
Source: PLoS Comput Biol. 2012 Nov 8;8(11):e1002767. doi: 10.1371/journal.pcbi.1002767 (PMC3493490; doi:10.1371/journal.pcbi.1002767)
Supplement: Text S1 — Documentation of the R-package in pdf format. (PDF) [file pcbi.1002767.s002.pdf]

Package ‘regoes12pcb’

October 8, 2012

Type Package

Title Supplementary online material accompanying Regoes RR, PLoS Comp Biol (2012)

Version 1.0

Date 2012-10-05

Author Roland R Regoes

Maintainer The package is not maintained, but questions can be sent to Roland R Regoes
`<roland.regoes@env.ethz.ch>`

Description This package contains the datasets, likelihoods and power analysis functions used in Regoes RR, PLoS Comp Biol (2012).

License GPL (>= 2)

LazyLoad yes

R topics documented:

regoes12pcb-package 2
fit.function 3
likelihoods.regoes12pcb 4
power.analysis 6
profile.likelihoods.regoes12pcb 7
rldc.data 9
sim.rldc 11

Index **13**

`regoes12pcb-package`
Supplementary online material accompanying Regoes RR, PLoS Comp Biol (2012)

Description

This package contains the repeated low-dose challenge data, likelihood and power analysis functions used in Regoes RR, PLoS Comp Biol (2012).

Details

This material is provided mostly for the sake of transparency. The functions were developed for the specific research questions and datasets that I analyzed in Regoes RR, PLoS Comp Biol (2012). They may not work beyond this restricted field of application.

I tried to document the functions as well as I could. However, documentation is only rudimentary, and will remain so because the package will not be updated.

For a summary of the data included in this package see `rldc.data`.

For a summary of the likelihood functions see `likelihoods.regoes12pcb`

See `power.analysis` for documentation of the power analysis function.

Author(s)

Roland R Regoes <roland.regoes@env.ethz.ch>

The package is not maintained. It is provided as supplementary online material to the paper Regoes RR, PLoS Comp Biol (2012).

References

Regoes RR (2012). The role of exposure history on HIV acquisition: insights from repeated low-dose challenge studies. PLoS Comp Biol.

See Also

`rldc.data` `likelihoods.regoes12pcb` `fit.function` `sim.rldc` `power.analysis`

Examples

```
## To fit the geometric infection model to the challenge data
## by Ellenberger et al, Virology (2006):
data(ellenberger06v)
fit.function(study="ellenberger06v", model="geom")

## To calculate the log likelihood of the heterogeneous
## susceptibility model with p.inf=0.2, v.inf=0.1 for the data
## by Wilson et al, J Virol (2006)
data(wilson06jv)
```

```
log.likelihood.frailty2(p.inf=0.2, v.inf=0.1,
                        challenges=wilson06jv$challenges.received,
                        infected=wilson06jv$infected)

## To generate challenge data on the basis of the geometric
## infection model for 10 animals, each challenged at most 20 times:
sim.rldc(n.animals = 10, n.chall.max = rep(20, 20),
         model = "geometric", model.pars = c(p.inf = 0.2))

## To assess the power to detect immunization in an experiment
## involving 20 animals that are challenged at most 20
## times each:
power.analysis(n.expt = 100, n.animals = 20, n.chall.max = rep(20,20),
               model.true = "immunization",
               model.pars = c(p.inf1 = 0.8, p.inf2 = 0.2),
               model.1 = "geometric", model.2 = "immunization")
```

`fit.function` *Fitting various models to repeated low dose challenge data.*

Description

Function that fits the geometric, immune priming, or heterogeneous susceptibility model to repeated low dose challenge data. This function was used in Regoes RR, PLoS Comp Biol (2012).

Usage

```
fit.function(study = "ellenberger06v",
             model = "geom", CI.type = "profile.ll")
```

Arguments

`study`	character string with the name of the data set to be analyzed: "ellenberger06v", "wilson06jv", "wilson09jv", "garciaLerma08pm", "hansen09nm", "hansen11n", "letvin11stm.SIVmac251", or "letvin11stm.SIVsmE660".
`model`	character string indicating the model to be fit. Possibilities are: "geom", "immune.1", "immune.2", "immune.3", "immune.inc", or "frailty2".
`CI.type`	character string indicating the type of confidence interval to calculate. Possibilities are "profile.ll" (the appropriate method), or the over-confident "fisher.info".

Value

Named vector with log.likelihood, parameter estimates, and upper and lower bounds of their 95% confidence intervals.

Note

Do not forget to load the dataset to be fitted, e.g. `data(wilson06jv)`.

Author(s)

Roland R Regoes <roland.regoes@env.ethz.ch>

References

Regoes RR (2012). The role of exposure history on HIV acquisition: insights from repeated low-dose challenge studies. PLoS Comp Biol.

See Also

rldc.data, likelihoods.regoes12pcb

Examples

```
## To fit the geometric infection model to the challenge data
## by Ellenberger et al, Virology (2006):
data(ellenberger06v)
fit.function(study="ellenberger06v", model="geom")
```

`likelihoods.regoes12pcb` *Likelihood functions*

Description

Likelihood functions for the geometric infection model, the immune priming models, and the heterogeneous susceptibility model defined in Regoes RR, PLoS Comp Biol (2012). For details please consult the article.

Usage

```
log.likelihood.geom.0(p.inf, challenges, infected)

log.likelihood.immune.l(p.inf1, p.inf2, l,
                        challenges, infected,
                        only.ll = FALSE)

log.likelihood.immune.inc(p.inf1, eps,
                          challenges, infected,
                          only.ll=FALSE)

log.likelihood.frailty2(p.inf, v.inf,
                        challenges, infected,
                        only.ll = FALSE)
```

Arguments

`p.inf`	scalar between 0 and 1 denoting the infection probability (geometric infection model), or the mean infection probability (heterogeneous susceptibility model).
`p.inf1`	scalar between 0 and 1 denoting the initial infection probability in the immune priming models.
`p.inf2`	scalar between 0 and 1 denoting the infection probability after the the lth challenge.
`l`	integer indicating the number of challenges after which susceptibility jumps from p.inf1 to p.inf2. Only relevant for the jump variant of the immune priming model.
`eps`	small scalar that can be positive or negative denoting the change in the infection probability from challenge to challenge.
`v.inf`	scalar denoting the variance in infection probability across animals. Its value is between 0 and (1-p.inf)*p.inf.
`challenges`	vector of integers indicating the number of challenges each animal received.
`infected`	logical vector indicating the infection status of each animal.
`only.ll`	logical. If TRUE gradient is not returned.

Value

A list with elements:

`ll`	the log likelihood
`gr`	a numerical vector containing the derivatives with respect to the model parameters.
`hessian`	only for the geometric infection model. For this model the hessian is a scalar.

Author(s)

Roland R Regoes <roland.regoes@env.ethz.ch>

References

Regoes RR (2012). The role of exposure history on HIV acquisition: insights from repeated low-dose challenge studies. PLoS Comp Biol.

See Also

`fit.function` uses these likelihoods. `rldc.data` lists datasets that can be feed to the likelihood functions.

Examples

```
data(wilson06jv)
log.likelihood.geom.0(p.inf=0.2,
                        challenges=wilson06jv$challenges.received,
                        infected=wilson06jv$infected)
```

power.analysis *Power to detect immunization or variation in susceptibility across animals in repeated low-dose challenge data.*

Description

This function estimates the statistical power to detect immunization effects or heterogeneous susceptibility in repeated low-dose challenge data. It was used in Regoes RR, PLoS Comp Biol (2012)

Usage

```
power.analysis(n.expt = 100, n.animals = 20, n.chall.max = rep(20, 20),
               model.true = "immunization",
               model.pars = c(p.inf1 = 0.8, p.inf2 = 0.2),
               model.1 = "geometric", model.2 = "immunization",
               output.fits = FALSE, output.not.converged = FALSE,
               output.dots = TRUE)
```

Arguments

`n.expt` number of in silico experiments to perform.

`n.animals` number of animals in the experiment.

`n.chall.max` integer vector of length `n.animals` containing the maximum number of challenges applied to each animal.

`model.true` character string defining the true model. Possible are "geometric", "immunization", or "frailty2". (This is passed on to `sim.rldc`.)

`model.pars` appropriately named vector with the parameters of the true model:

```
model.true="geometric"      ->  model.pars=c(p.inf = xxx)
model.true="immunization"   ->  model.pars=c(p.inf1 = xxx, p.inf2 = yyy)
model.true="frailty2"       ->  model.pars=c(p.inf = xxx, v.inf = yyy)
```

For appropriate parameter ranges see `likelihoods.regoes12pcb`.

`model.1` character string defining the first model to fit. Possible are "geometric", "immunization", or "frailty2". The first model is compared to the fit of the second model by a likelihood ratio test.

`model.2` character string defining the second model to fit. Possible are "geometric", "immunization", or "frailty2". The first model is compared to the fit of the second model by a likelihood ratio test.

`output.fits` logical. If TRUE fits are returned. Only for diagnostic purposes and low `n.expt`.

`output.not.converged`
logical. If TRUE prints data and fit details of non-converged fits. Only for diagnostic purposes.

`output.dots` logical. If TRUE the function prints "*" for experiments that establish a significant difference between the fits of `model.1` and `model.2`, "." if there is no significant difference, and "x" or "?" if there were convergence problems.

Value

Returns a list with the power estimate and the fraction of fits of the second model that converged. If `output.fits=TRUE` the fits are also returned.

Author(s)

Roland R Regoes <roland.regoes@env.ethz.ch>

References

Regoes RR (2012). The role of exposure history on HIV acquisition: insights from repeated low-dose challenge studies. PLoS Comp Biol.

See Also

The in silico repeated low-dose challenge data are generated with the function `sim.rldc`.

Examples

```
## To assess the power to detect immunization in an experiment
## involving 20 animals that are challenged at most 20
## times each:
power.analysis(n.expt = 100, n.animals = 20, n.chall.max = rep(20,20),
               model.true = "immunization",
               model.pars = c(p.inf1 = 0.8, p.inf2 = 0.2),
               model.1 = "geometric", model.2 = "immunization")
```

`profile.likelihoods.regoes12pcb` *Profile likelihood functions*

Description

Profile likelihood functions for each parameter of the geometric infection, immune priming, and heterogeneous susceptibility models. This is call by `fit.function` to calculate confidence intervals.

Usage

```
log.likelihood.immune.l.prof.p.inf1(p.inf1, l = 1,
                                    challenges, infected,
                                    output.nuisance.par = FALSE)

log.likelihood.immune.l.prof.p.inf2(p.inf2, l = 1,
                                    challenges, infected,
                                    output.nuisance.par = FALSE)

log.likelihood.immune.inc.prof.p.inf1(p.inf1,
                                      challenges, infected,
                                      output.nuisance.par = FALSE)

log.likelihood.immune.inc.prof.eps(eps,
                                   challenges, infected,
                                   output.nuisance.par = FALSE)

log.likelihood.frailty2.prof.p.inf(p.inf,
                                   challenges, infected,
                                   output.nuisance.par = FALSE)

log.likelihood.frailty2.prof.v.inf(v.inf,
                                   challenges, infected,
                                   output.nuisance.par = FALSE)
```

Arguments

`p.inf`	scalar between 0 and 1 denoting the infection probability (geometric infection model), or the mean infection probability (heterogeneous susceptibility model).
`p.inf1`	scalar between 0 and 1 denoting the initial infection probability in the immune priming models.
`p.inf2`	scalar between 0 and 1 denoting the infection probability after the the lth challenge.
`l`	integer indicating the number of challenges after which susceptibility jumps from p.inf1 to p.inf2. Only relevant for the jump variant of the immune priming model.
`eps`	small scalar that can be positive or negative denoting the change in the infection probability from challenge to challenge.
`v.inf`	scalar denoting the variance in infection probability across animals. Its value is between 0 and (1-p.inf)*p.inf.
`challenges`	vector of integers indicating the number of challenges each animal received.
`infected`	logical vector indicating the infection status of each animal.
`output.nuisance.par`	logical. If set to TRUE, the value of nuisance parameter maximizing the profile likelihood is returned also.

Value

The value of the log profile likelihood, or, if `output.nuisance.par==TRUE` a list with likelihood and nuisance parameter.

Author(s)

Roland R Regoes <roland.regoes@env.ethz.ch>

References

Severini TA (2000). Likelihood Methods in Statistics. Oxford University Press, 123–129 pp.

Regoes RR (2012). The role of exposure history on HIV acquisition: insights from repeated low-dose challenge studies. PLoS Comp Biol.

See Also

See `likelihoods.regoes12pcb` for the likelihoods. This function is used by `fit.function`

`rldc.data` *Repeated low dose challenge data*

Description

Repeated low dose challenge data from various sources. The datasets contain the number of challenges each animal received and information on each animals final infection status.

Usage

```
data(ellenberger06v)

data(wilson06jv)

data(wilson09jv)

data(garciaLerma08pm)

data(hansen09nm)

data(hansen11n)

data(letvin11stm.SIVmac251)

data(letvin11stm.SIVsmE660)
```

Format

Data frames with the following columns:

`Treatment` a factor with only one level `control`.

`AnimalID` unique identifier of each animal

`challenges.received` a numeric vector indicating the number of challenges received by each animal.

`infected` a logical vector indicating the final infection status of each animal

Details

The data were collected from the publications cited below, and analyzed in Regoes RR (2012) PLoS Comp Biol.

In some instances, tables with the data were available in the printed versions of the articles. In most cases, however, the data were read off from Kaplan-Meier curves in the papers or their accompanying supplementary material in combination with information in the text.

The data from Hansen et al, Nat Med (2009), were read off the virus load time courses in their Figure 4c. From that Figure, virus loads profiles from 15 control monkeys can be identified. This is inconsistent with the statements of the authors that there were 16 control monkeys. Although I asked the authors about this multiple times they did not clarify this inconsistency.

References

Ellenberger D, Otten RA, Li B, Aidoo M, Rodriguez IV, et al. (2006) HIV-1 DNA/MVA vaccination reduces the per exposure probability of infection during repeated mucosal SHIV challenges. Virology 352: 216-225.

Garcia-Lerma JG, Otten RA, Qari SH, Jackson E, Cong ME, et al. (2008) Prevention of rectal SHIV transmission in macaques by daily or intermittent prophylaxis with emtricitabine and tenofovir. PLoS Med 5: e28.

Hansen SG, Vieville C, Whizin N, Coyne-Johnson L, Siess DC, et al. (2009) Effector memory T cell responses are associated with protection of rhesus monkeys from mucosal simian immunodeficiency virus challenge. Nat Med 15: 293-9.

Hansen SG, Ford JC, Lewis MS, Ventura AB, Hughes CM, et al. (2011) Profound early control of highly pathogenic SIV by an effector memory T-cell vaccine. Nature 473: 523-7.

Letvin NL, Rao SS, Montefiori DC, Seaman MS, Sun Y, et al. (2011) Immune and Genetic Correlates of Vaccine Protection Against Mucosal Infection by SIV in Monkeys. Sci Transl Med 3: 81ra36.

Wilson NA, Reed J, Napoe GS, Piaskowski S, Szymanski A, et al. (2006) Vaccine-induced cellular immune responses reduce plasma viral concentrations after repeated low-dose challenge with pathogenic simian immunodeficiency virus SIVmac239. J Virol 80: 5875-85.

Wilson NA, Keele BF, Reed JS, Piaskowski SM, MacNair CE, et al. (2009) Vaccine-induced cellular responses control simian immunodeficiency virus replication after heterologous challenge. J Virol 83: 6508-21.

Examples

```
data(ellenberger06v)
ellenberger06v
```

`sim.rldc` *Generating repeated low dose challenge data*

Description

Function that generates repeated low dose challenge data according to the geometric, immune priming, or heterogeneous susceptibility models defined in Regoes RR, PLoS Comp Biol (2012).

Usage

```
sim.rldc(n.animals = 20, n.chall.max = rep(20, 20),
         model = "geometric", model.pars = c(p.inf = 0.2),
         print.comments = TRUE)
```

Arguments

`n.animals`	number of animals in the experiment.
`n.chall.max`	integer vector of length `n.animals` containing the maximum number of challenges applied to each animal.
`model`	character string defining the true model. Possible are "geometric", "immunization", or "frailty2".
`model.pars`	appropriately named vector with the parameters of the true model. E.g. for `model.true = "immunization"` we need `model.pars = c(p.inf1 = 0.8, p.inf2 = 0.2)`. The names of `model.pars` can be seen in the `likelihoods.regoes12pcb`.
`print.comments`	logical. If `TRUE` model and parameter info printed in the beginning of the output.

Value

Data frame with columns entitled: "AnimalID", "challenges.received", and "infected".

Author(s)

Roland R Regoes <roland.regoes@env.ethz.ch>

References

Regoes RR (2012). The role of exposure history on HIV acquisition: insights from repeated low-dose challenge studies. PLoS Comp Biol.

See Also

This function is used by `power.analysis`

Examples

```
sim.rldc(n.animals = 20, n.chall.max = rep(20, 20),
         model = "geometric", model.pars = c(p.inf = 0.2),
         print.comments = TRUE)
```

Index

*Topic **regoes12pcb**
fit.function, 3
likelihoods.regoes12pcb, 4
power.analysis, 5
profile.likelihoods.regoes12pcb, 7
regoes12pcb-package, 1
rldc.data, 9
sim.rldc, 10

ellenberger06v *(rldc.data)*, 9

fit.function, *2*, 3, *5*, *7*, *8*

garciaLerma08pm *(rldc.data)*, 9

hansen09nm *(rldc.data)*, 9
hansen11n *(rldc.data)*, 9

letvin11stm.SIVmac251 *(rldc.data)*, 9
letvin11stm.SIVsmE660 *(rldc.data)*, 9
likelihoods.regoes12pcb, *2*, *3*, 4, *8*
log.likelihood.frailty2 *(likelihoods.regoes12pcb)*, 4
log.likelihood.frailty2.prof.p.inf *(profile.likelihoods.regoes12pcb)*, 7
log.likelihood.frailty2.prof.v.inf *(profile.likelihoods.regoes12pcb)*, 7
log.likelihood.geom *(likelihoods.regoes12pcb)*, 4
log.likelihood.immune.inc *(likelihoods.regoes12pcb)*, 4
log.likelihood.immune.inc.prof.eps *(profile.likelihoods.regoes12pcb)*, 7
log.likelihood.immune.inc.prof.p.inf1 *(profile.likelihoods.regoes12pcb)*, 7
log.likelihood.immune.l *(likelihoods.regoes12pcb)*, 4
log.likelihood.immune.l.prof.p.inf1 *(profile.likelihoods.regoes12pcb)*, 7
log.likelihood.immune.l.prof.p.inf2 *(profile.likelihoods.regoes12pcb)*, 7
log.likelihoods.regoes12pcb *(likelihoods.regoes12pcb)*, 4

power.analysis, *2*, 5
profile.likelihoods.regoes12pcb, 7

regoes12pcb *(regoes12pcb-package)*, 1
regoes12pcb-package, 1
rldc.data, *2*, *3*, *5*, 9

sim.rldc, *2*, *7*, 10

wilson06jv *(rldc.data)*, 9
wilson09jv *(rldc.data)*, 9
